# Nanomedicine-driven neuropathic pain relief in a rat model is associated with macrophage polarity and mast cell activation

**DOI:** 10.1186/s40478-019-0762-y

**Published:** 2019-07-05

**Authors:** Muzamil Saleem, Brooke Deal, Emily Nehl, Jelena M. Janjic, John A. Pollock

**Affiliations:** 1 Department of Biological Sciences, Duquesne University, Pittsburgh, PA USA; 2 Graduate School of Pharmacy, Duquesne University, Pittsburgh, PA USA; 3 Chronic Pain Research Consortium, Duquesne University, Pittsburgh, PA USA; 4 Jacobs School of Medicine and Biomedical Sciences, University of Buffalo, Buffalo, NY USA

**Keywords:** Inflammation, Nanomedicine, Neuropathic pain, Chronic pain, Macrophage, Mast cell

## Abstract

We explored the immune neuropathology underlying multi-day relief from neuropathic pain in a rat model initiated at the sciatic nerve, by using a nanoemulsion-based nanomedicine as a biological probe. The nanomedicine is theranostic: both therapeutic (containing celecoxib drug) and diagnostic (containing near-infrared fluorescent (NIRF) dye) and is small enough to be phagocytosed by circulating monocytes. We show that pain-like behavior reaches a plateau of maximum hypersensitivity 8 days post-surgery, and is the rationale for intravenous delivery at this time-point. Pain relief is evident within 24 h, lasting approximately 6 days. The ipsilateral sciatic nerve and associated L4 and L5 dorsal root ganglia (DRG) tissue of both nanomedicine and control (nanoemulsion without drug) treated animals was investigated by immunofluorescence and confocal microscopy at the peak of pain relief (day-12 post-surgery), and when pain-like hypersensitivity returns (day-18 post-surgery). At day-12, a significant reduction of infiltrating macrophages, mast cells and mast cell degranulation was observed at the sciatic nerve following treatment. In the DRG, there was no effect of treatment at both day-12 and day-18. Conversely, at the DRG, there is a significant increase in macrophage infiltration and mast cell degranulation at day-18. The treatment effect on immune pathology in the sciatic nerve was investigated further by assessing the expression of macrophage cyclooxygenase-2 (COX-2)—the drug target—and extracellular prostaglandin E2 (PGE2), as well as the proportion of M1 (pro-inflammatory) and M2 (anti-inflammatory) macrophages. At day-12, there is a significant reduction of COX-2 positive macrophages, extracellular PGE2, and a striking reversal of macrophage polarity. At day-18, these measures revert to levels observed in control-treated animals. Here we present a new paradigm of immune neuropathology research, by employing a nanomedicine to target a mechanism of neuropathic pain—resulting in long-lasting pain relief--whilst revealing novel immune pathology at the injured nerve and associated DRG.

**Electronic supplementary material:**

The online version of this article (10.1186/s40478-019-0762-y) contains supplementary material, which is available to authorized users.

## Introduction

Pain remains the most pervasive reason for medical visits worldwide and affects more people than cancer, heart disease, and diabetes combined (NIH, 2010). It is a burden to society, as well as the wider economy [19] and afflicts approximately 20% of the world’s population [22]. Pain is ineffectively managed, largely because the underlying neuropathology is poorly understood, and because it has been mischaracterized by health care systems solely as a symptom of other diseases. When pain transitions from acute to chronic, it becomes a neurobiological disease in its own right [52] as defined by the World Health Organization (WHO, 2017). Traditionally, efforts to understand the mechanisms underlying chronic pain have centered on neuronal plasticity [4, 15]: nociceptor sensitization in the peripheral nervous system [20, 54], and sensitization of pain circuits in the central nervous system [69]. In the last two decades, it has been shown that there is a non-neuronal input—largely from the immune system—that contributes to nociceptor sensitization [69]. This includes neuroinflammation, which results from a number of insults, such as injury, neurodegeneration, autoimmunity, and infection [34].

We sought to further our understanding of the immune-cell pathology underlying neuropathic pain by utilizing the chronic constriction injury (CCI) rat model [7]. In this model, an inflammatory response is produced by loosely tied chromic gut ligatures on the right sciatic nerve, causing swelling and subsequent constriction of the nerve. The inflamed nerve is infiltrated by a complex milieu of immune cells, inflammatory mediators [64] and signaling molecules, resulting in nociceptor sensitization, and causing persistent pain [21]. This inflammatory response is largely driven by the infiltration of macrophages [33, 43], which express the cyclooxygenase-2 (COX-2) enzyme. COX-2 synthesizes PGE2, and in addition, the macrophage releases various other cytokines and chemokines [64]. Rats exhibit progressive hypersensitive pain-like behavior reaching a maximum approximately 8 days following surgery [31, 63, 64].

In earlier studies, Janjic and colleagues have designed and developed theranostic nanoemulsions that specifically target macrophages via phagocytosis by circulating monocytes, and subsequent natural migration and accumulation at sites of inflammation [47], including injured nerve [31]. In this paradigm, we are also able to image the extent of neuroinflammation by detecting a near-infrared fluorescent (NIRF) signal from the nanoemulsion-contained DiR fluorescent dye in live animals [31, 63]. We have previously described the formulation of drug loaded perfluorocarbon nanoemulsions [31, 50]. Briefly, oil dispersion is formed in aqueous medium using high energy processing such as microfluidization. Lipophilic drug is dissolved in the oil core of the nanoemulsion droplet. The nanoemulsion is loaded with the nonsteroidal anti-inflammatory drug (NSAID), celecoxib, and is designed to target and inhibit COX-2 activity in monocytes. The rationale described previously by Janjic and colleagues [31, 47, 50] for drug-loading nanoemulsion droplets with celecoxib is to directly attenuate the COX-2 enzyme, which synthesizes PGE2, a potent proinflammatory mediator. PGE2 perpetuates the neuroinflammation that sensitizes nociceptors, leading to neuropathic pain. Celecoxib directly binds to the active site on the COX-2 enzyme, thereby blocking the synthesis of PGE2 [23]. We have previously shown that nanomedicine treatment reverses pain-like behavior and reduces inflammation in a neuropathic pain model in rats [31] and in an inflammation model in mice [47].

We designed an experiment with the aim of exploring key aspects of macrophage and mast cell neuropathology whilst pivoting at key events on a timeline of neuropathic pain: a state where the animal exhibits pain-like behavior, a state where the animal experiences peak pain-relief (day-12 post-surgery)—as a result of nanomedicine treatment [31]—and a state when the animal returns to pain-like behavior (day-18 post-surgery). Testing groups of CCI rats are intravenously administered with nanomedicine or vehicle treatment (nanoemulsion without celecoxib) 8-days post-surgery—due to animals showing peak pain-like behavior at this time point. A control group of animals undergoes a sham CCI surgery. Key in the design of the nanomedicine is the capability of visualizing macrophages [30, 35, 39, 46–49] that have phagocytosed the nanoemulsion (~ 140 nm per droplet). In this way, the theranostic nanomedicine—that is both ‘therapeutic’ and ‘diagnostic’—serves as a biological probe to both influence and/or label aspects of the underlying neuropathology.

Pivoting on the behavioral states of *pain*, *pain relief*, and *return to pain* as compared to control animals, we first present the extent of inflammation measured by NIRF imaging of the live animal and then report on our findings from histological analysis of sciatic nerve and its associated L4 and L5 DRG. Using confocal microscopy, the quantity of infiltrating macrophages is measured—marked by anti-CD68 antibody, a pan-macrophage marker [25]—in the injured sciatic nerve and associated L4 and L5 DRG neurons.

Along with macrophages, resident mast cells constitute another major cell group involved in the inflammatory response. They are found close to nociceptive neuron cell bodies [18]—as well as in a progenitor form in the blood circulation—and are associated with a number of clinical pain disorders [10]. With respect to the inflammatory component of neuropathic pain, mast cells provide an input to cause neurogenic inflammation, which propagates along afferent nociceptors via the release of substance P [10]. Mast cells are filled almost entirely with secretory granules and contain a vast assortment of inflammatory mediators and other bioactive molecules such as cytokines, lysosomal hydrolases and proteases [68]. In this study, mast cell count and extent of degranulation in the sciatic nerve and associated DRG is investigated by labeling with an antibody targeted to mast cell protease 1 (Mcpt-1). Mcpt1 constitutes a major component of the secretory granules released by mast cells during an inflammatory response and is specific to this cell type. The cellular expression of Mcpt1 labels mast cells, and when expressed extracellularly, indicates that a mast cell has been activated and degranulation has occurred. We present data reporting on the numbers of mast cells, and the extent of their degranulation, at the sciatic nerve—and the associated DRG—following nanomedicine treatment.

The treatment effect observed at the sciatic nerve was probed further by investigating individual macrophages to reveal details of their inflammatory phenotype, in addition to whether they are positive for nanomedicine NIRF signal. We also report on the expression of COX-2 in macrophages and their release of PGE2 in the milieu of the injured sciatic nerve tissue. Macrophages play a dual role in damaged tissues—a subset performs inflammatory functions and are termed M1 pro-inflammatory macrophages, while those that are anti-inflammatory effectors, promoting tissue repair, are termed M2 macrophages [27, 44]. A recent membrane proteome study [6] was able to discriminate M1 (pro-inflammatory) and M2 (anti-inflammatory) macrophages with high precision by their expression of the costimulatory protein, Cluster of Differentiation 40 (CD40), and transferrin receptor (TFRC) respectively. These markers were thus used to investigate the percentage of M1 and M2 macrophages in the injured sciatic nerve. Our present paper details the relative macrophage polarity in the context of treatment, and pain-like behavior. We also report on the presence of CD68-positive multinucleated giant cells (MGCs) in the injured nerve—cells formed from the fusion of their M2 polarized macrophage precursors. MGCs have been reported to enhance the removal of debris from tissues [51], aiding tissue regeneration.

This paper proposes a mechanism for nanomedicine-driven pain relief in a rat model of neuropathic pain. We present the interplay of COX-2 attenuation, PGE2 production and the resulting effect on macrophage polarity. In addition, macrophage infiltration to the injured nerve is investigated, as well as the formation of multinucleated giant cells, which contribute to tissue regeneration and repair. The numbers of mast cells localized in the injured nerve and DRG and the extent of their degranulation is investigated. Based on patterns of macrophage infiltration, changes in their phenotype, and levels of mast cell degranulation, we suggest a mechanism that could underscore the return to pain-like behavior when the treatment effect has subsided. Taken together, this paper provides key insights into the neuropathology underlying neuropathic pain, by utilizing a nanomedicine as a biological probe. Having the ability to track patterns of macrophage infiltration both during and after long-lasting pain relief, we demonstrate a novel research paradigm that could be useful in more precisely elucidating the neuropathology underlying diseases with an immune component.

## Materials and methods

### Animal testing groups and chronic constriction injury model of neuropathic pain

The CCI [7] animal model was used to induce neuropathic pain in rats as previously described [31]. Briefly, animals were divided into three groups; CCI rats administered with nanomedicine containing no drug (DF-NE), CCI rats administered with nanomedicine containing drug (CXB-NE) and sham surgery rats. We have previously shown biodistribution data of vehicle nanoemulsion in rats that undergo sham surgery [63], indicating no NIRF signal accumulation at the site of the ipsilateral sciatic nerve. Under isoflurane anesthesia, the skin was incised and the biceps femoris muscles separated to expose the sciatic nerve. Chromic gut suture was used to tie four ligatures approximately 1 mm apart around the common sciatic nerve. Care was taken to ensure the ligatures were neither tight nor loose. The biceps femoris muscles were closed using chromic gut suture followed by skin closure using stainless steel wound clips. An identical surgery was performed on animals in the sham CCI group, without ligatures being tied to the sciatic nerve.

### Pain-like behavior testing

**Fig. 1:**
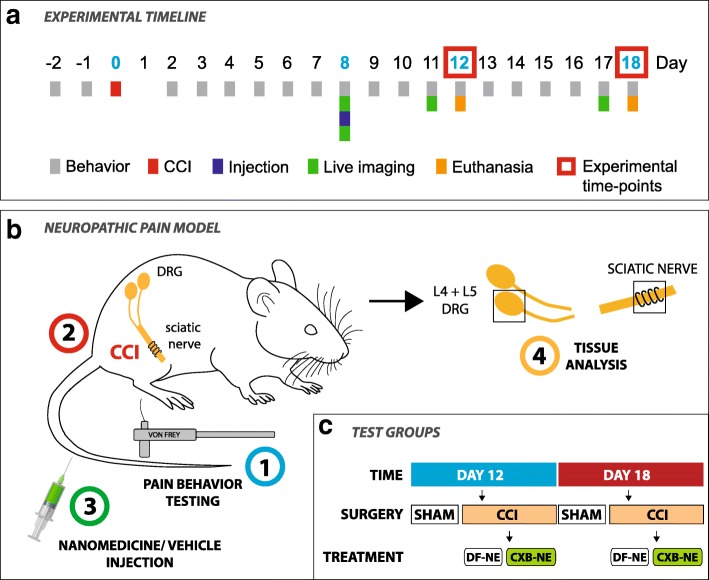
Summary of the experimental timeline, neuropathic pain model and testing groups. **a** A daily timeline indicates the procedures that a rat undergoes for both the 12-day test group and the 18-day test group. Mechanical stimulus-evoked pain-like hypersensitivity testing (**b1**), surgery (**b2**), tail vein injection of treatment (**b3**), live-animal NIRF imaging, and recovery of tissue for analysis (**b4**). Sciatic nerve and DRG (RL4 and RL5) tissue is collected from the animal following euthanasia and perfusion-fixation on day-12 and day-18 (**a**, **b4**). Test groups are split into time-points of day-12 and day-18, each with groups of CCI and sham surgery rats (**c**). The surgery groups are further divided into rats that are treated with drug-free nanomedicine (DF-NE) and those treated with celecoxib nanomedicine (CXB-NE) (**c**)

To assess mechanical allodynia—indicative of pain-like hypersensitivity—von Frey filaments were applied to the plantar surface of the hind-paw. The threshold force at which rats withdrew their paws 50% of the time was determined using the up-down method and is adapted from a statistical analysis used to determine median lethal dose (LD50) [9, 17]. Testing was performed at the same time of day during their light cycle. Rats were acclimated for 15 min in the testing apparatus: perspex chambers with a wire mesh floor that allows access to the paws. Baseline testing was carried out for two consecutive days before surgery. The rats were rested for 1 day following surgery, after which behavioral testing resumed a day later, and on consecutive days, ceasing on the day-12 or day-18 following surgery (summarized in Fig. 1a).

### Development, preparation, and testing of NIRF-labelled, celecoxib-loaded nanomedicine

A perfluorocarbon-based nanoemulsion was formulated with a DiR near-infrared cyanine dye (ThermoFisher Scientific, Waltham, MA), and loaded with celecoxib as previously described [31, 50]. Colloidal stability of the nanoemulsion and its drug-loading potential was assessed to ensure quality control [31]. In vitro cell culture using the murine macrophage RAW 264.7 cell line (ATCC Lot #61524889, Manassas, VA) was used to assess cellular uptake of nanoemulsion [49], as well as the viability of the macrophages following incubation with different concentrations of nanoemulsion [31].

### Rat tail-vein injection of nanomedicine

The nanomedicine—celecoxib-loaded (CXB-NE) and drug-free (DF-NE)—was injected intravenously via the lateral tail vein of rats on day-8 post-surgery (Fig. 1a) as previously described [31, 60]. Injection of 300 μL of CXB-NE or DF-NE was performed with an intravenous catheter with a blood-flow indicator (Terumo, Tokyo, Japan). The single dose of celecoxib in the CXB-NE group was ~ 0.24 mg/kg. We selected this dose in a previous study [31] to be significantly lower than a reported effective oral dose administered twice daily for 10 days [61, 67]—thereby demonstrating the effectiveness of our nanomedicine approach. The successful injection was confirmed by both the blood indicator on the catheter as well as using a pre and post-injection NIRF image to make a quality assessment [60].

### NIRF imaging in live animals

The right and left thigh of anesthetized rats from CCI (DF-NE) and CCI (CXB-NE) groups were imaged with a preclinical fluorescence imager (LiCOR® Pearl Impulse from LI-COR Biosciences, Lincoln, NE) on day-11 and day-17 post-surgery, in day-12 and day-18 testing groups respectively. We have previously shown no NIRF signal at the site of the sciatic nerve in sham surgery animals [63], hence this group was not included in the in-vivo imaging investigation. The NIR dye in the nanomedicine that has accumulated in labeled monocytes/ tissue macrophages fluoresces in the scanner. Images in the fluorescent channel (785 nm excitation for 820 nm emission) and a white light channel (to capture an image of the body of the rat) are acquired and merged in the LiCOR Pearl Impulse Software (version 2.0) with linked look-up-tables (LUT) [63]. In order to avoid non-specific fluorescence in the abdominal and thoracic region that can be caused by certain foods [8], animals were given a controlled research diet throughout the procedure [63] (D10012G Research Diets, Inc. New Brunswick, NJ). Many plant-based diets given to rodents contain chlorophyll, which fluoresces naturally and can result in unreliable in-vivo NIRF imaging, therefore using a controlled diet that does not fluoresce is essential. NIRF images were analyzed using Image Studio Lite Software (LI-COR Biosciences, Lincoln, NE) as previously described [31, 63]. Briefly, a region of interest (ROI) was selected over the sciatic nerve. The relative fluorescence of each ROI was calculated by dividing the total fluorescence in the ROI by the area occupied by the region.

### Euthanasia

Following behavioral testing and in-vivo NIRF imaging, rats were euthanized under anesthesia with a 2 ml intraperitoneal injection of Euthasol (pentobarbital sodium and phenytoin sodium solution, Virbac AH, Inc., Fort Worth, TX). The animals were immediately perfused with 180 mL of cold 1X PBS followed by 180 mL of 4% paraformaldehyde 1X PBS solution, administered into the left ventricle of the heart, resulting in whole body fixation.

### Tissue processing

Each animal in the study is used for behavioral testing, in-vivo NIRF imaging and finally has their tissue processed for further immunohistochemical testing. Sciatic nerve tissue—and separately, L4 and L5 DRG tissue—was dissected on day-12 or day-18 post-surgery from the CCI (DF-NE) and CCI (CXB-NE) groups. We have previously shown that there is virtually no monocyte infiltration to the ipsilateral sciatic nerve of rats that have undergone sham surgery [63]—and additionally, no expression of COX-2 or PGE2 [31]. This provided the rationale for focusing the immunohistochemical analysis on tissue from CCI animals treated with DF-NE and those administered with CXB-NE. Tissue was post-fixed in 4% PFA (pH 7.4) in 1X PBS for 24 h at 4 °C and then transferred to 30% sucrose in 1X PBS, and stored at 4 °C until further processing. Tissue was prepared for sectioning by transferring to optimal cutting temperature (OCT) solution (Sakura, Torrance, CA) and frozen in a bath of isopentane maintained at a temperature of ~ − 55 to − 60 °C surrounded by dry ice. Frozen tissue was sectioned at a thickness of 20 μm and mounted on gelatin-coated slides (SouthernBiotech, Birmingham, AL). The slides were stored at − 20 °C until further processing.

### Immunofluorescence and nanomedicine NIRF detection

Slides were processed using the primary antibodies listed in Additional file 1: Table S1 at the stated dilutions prepared from manufacturer stock solutions. Double-staining was performed with two primary antibodies in each experiment. Appropriate secondary antibodies raised in different hosts were selected to target the host of the primary antibody in order to prevent nonspecific binding. Sections stored at − 20 °C were warmed on a slide warmer at 37 °C for 30 min. Sections were post-fixed in 4% paraformaldehyde in 1X PBS solution for 15 min and permeabilized for 10 min in 0.3% Triton X-100 detergent in 1X PBS. Tissue blocking of nonspecific staining was performed by incubating with BlockAid™ Blocking Solution (B10710, Thermo Fisher Scientific) for 1 h at room temperature. This solution was also used to prepare all working antibody solutions. The sections were incubated overnight at 4 °C with primary antibody prepared at the appropriate working dilution (Additional file 1: Table S1). The following day, sections were washed in 0.3% Triton X-100 detergent in 1X PBS and incubated in secondary antibody solution for 2 h at room temperature. Following washing in 0.3% Triton X-100 detergent in 1X PBS, sections were mounted using Prolong Diamond antifade reagent with DAPI (P36965, Thermo Fisher Scientific). The nanomedicine contains DiR so that a ‘double-stain’ experiment actually has four dyes in the tissue; DAPI (nuclei), two secondary-antibody conjugated fluorophores (Additional file 1: Table S2), and DiR (nanomedicine).

### Confocal microscopy and image analysis

All stained sections were scanned by the Nikon A1 confocal microscope equipped with six excitation solid-state diode lasers (405 nm, 440 nm, 488 nm, 514 nm, 561 nm, and 640 nm) and acquired with the Nikon NIS-Elements software. Confocal images for a comparative set were acquired with the same instrument settings (laser power, gain, etc.). Image analysis was performed with the FIJI distribution of ImageJ (version 1.52i) software. For cell analysis, images were acquired in multiple channels to capture a marker for the cell, an additional protein-of-interest, as well as the NIRF signal emitted from the nanomedicine. Regions-of-interest were drawn around individual cells in an image and fluorescence intensity (mean fluorescence/ area) in each channel was measured. For each experiment, a threshold of mean intensity/ area was allocated in relevant confocal imaging channels (e.g. protein of interest and nanomedicine NIRF) by sampling multiple images to discriminate cells positive for a protein-of-interest, from those that were negative for the protein-of-interest. Subsequently, the total cell count, those cells positive for a protein-of-interest, and those positive for nanomedicine was recorded. Next, it was determined which cells were both positive for the protein-of-interest and the nanomedicine NIRF signal. Particle analysis for Mcpt1 and extracellular PGE2 was performed by first applying a threshold to the image to select stained particles and cells. Next, a size discrimination threshold was applied to exclude the relatively larger cells—to leave behind particles—and a particle count was performed for each image.

### Statistical analysis

**Fig. 2:**
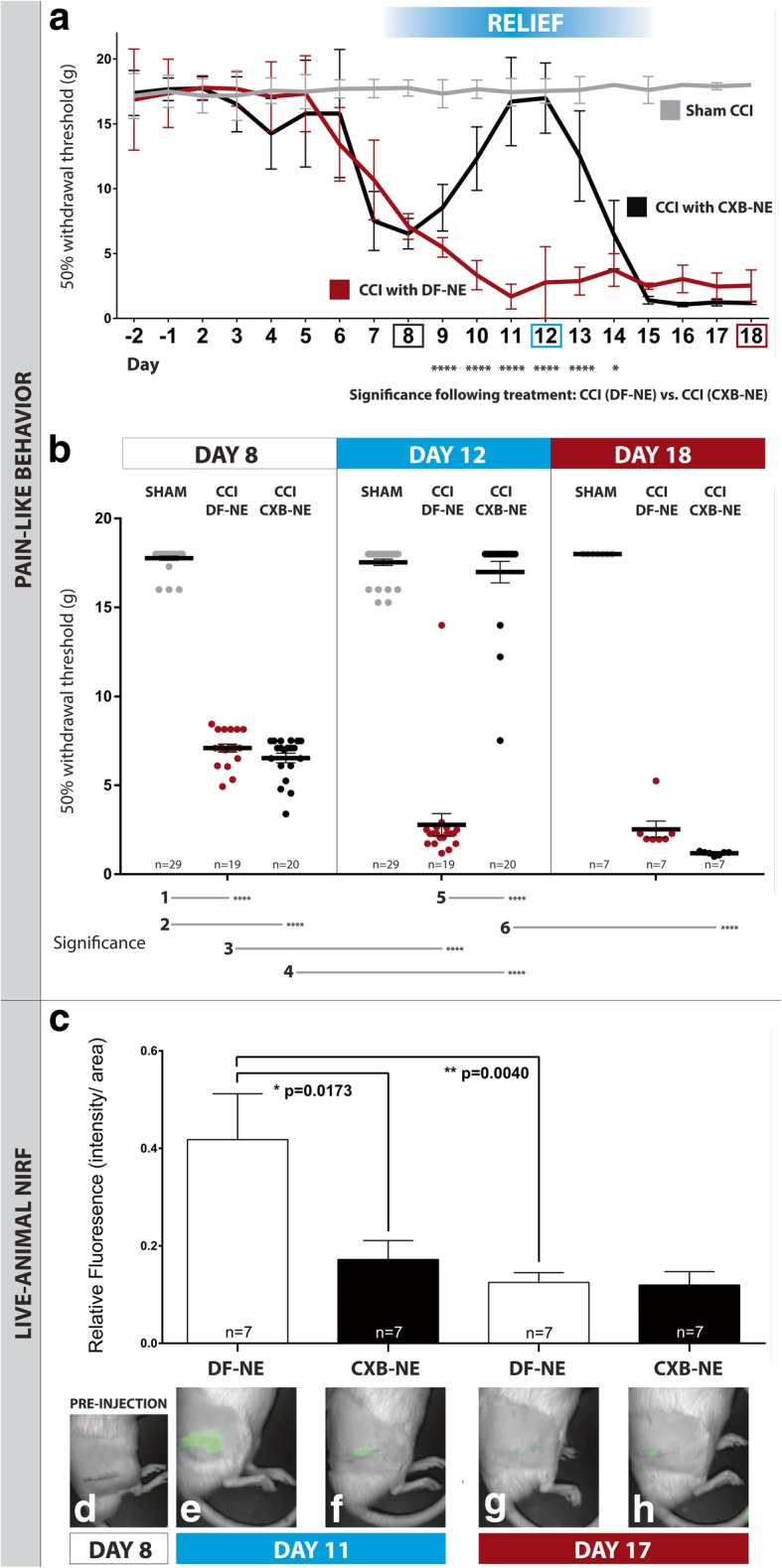
Mechanical stimulus-evoked pain-like hypersensitivity testing and live animal NIRF imaging. The manual up-down Von Frey test is performed on the days outlined in Fig. 1a to evaluate mechanical allodynia. Panel **a** shows results from daily testing, and panel **b** summarizes 50% withdrawal thresholds at day-8, day-12, and day-18. At day-8, both CCI animal groups are significantly more hypersensitive than sham CCI animals (**a** and **b**). Nanomedicine is injected after behavioral testing is completed on day-8. The CCI animals given CXB-NE show a significant reversal in withdrawal thresholds at day-12, a level similar to the sham CCI group (**a** and **b**). The reversal in pain-like hypersensitivity lasts for approximately 6 days (shown in **a**, day-9 to 14; ****p < .0001, *p = 0.0257, two-way ANOVA with Tukey’s posthoc test) and by day-18 the withdrawal threshold lowers back to a level indicative of a chronic pain state (**a** and **b**). All associated data analysis can be found here [57]: 10.6084/m9.figshare.8287823.v1. Whole-body live-animal NIRF imaging is performed on day 11 and day 17, the evening before day-12 and day-18 animals respectively are euthanized, and perfusion fixed. In the live animals at day-11, there is a significant decrease in NIRF signal in CCI animals given CXB-NE (**c** and **f**) compared to animals administered with DF-NE (**c** and **e**). The animals show no fluorescence at day-8, prior to injection (**d**). At day-17, NIRF signal at the site of the ipsilateral sciatic nerve is significantly decreased in the CCI group given DF-NE (**g**). A similar level of NIRF signal is observed at day-17 in the CCI group given CXB-NE (**h**). Pain behavior data is represented as mean ± SD (*n* = 7–29 animals; *p < .05, ****p < .0001, one-way ANOVA with Tukey’s posthoc test). In vivo imaging data is represented as mean ± SEM (*n* = 7 animals; *p < .05, **p < .01, one-way ANOVA with Tukey’s post hoc test)

Following testing for pain-like behavior, the 50% paw withdrawal threshold was calculated, and treatment groups were analyzed by two-way ANOVA across day-8, day-12 and day-18 time-points (Fig. 2b). Additionally, a one-way ANOVA was performed separately for each time-point (Fig. 2a). The Tukey’s post hoc test for multiple comparisons of group means was performed following the one-way and two-way ANOVA analyses. The confidence interval is 95% and data are presented as mean ± SD. Relative NIRF data are analyzed by one-way ANOVA, with a Tukey’s post hoc test to test multiple comparisons of group means. The confidence interval is set at 95%. Data are presented as mean ± SEM. Cell and particle count data are analyzed by one-way ANOVA with Tukey’s post hoc test to test multiple comparisons of group means. A confidence interval of 95% was set. Data are presented as mean ± SEM. The ANOVA analyses were performed on the GraphPad Prism 6 statistical software program. In order to compare the differences between percentages of proteins-of-interest colocalized with macrophages between test groups, Pearson Chi-Square, and Fisher’s exact tests were utilized and performed on IBM SPSS Statistics 25 software. A confidence interval of 95% was set, and a Fisher’s exact test *p*-value is computed.

## Results

### Nanomedicine treatment relieves pain-like hypersensitivity for ~ 6 days

Manual Von Frey mechanical allodynia testing was performed to measure pain-like hypersensitivity in rats modeled with neuropathic pain. A paw withdrawal threshold is calculated; a lower value infers a higher level of pain-like hypersensitivity. Baseline paw withdrawal thresholds were measured on two consecutive days preceding CCI and sham CCI surgery—where no statistical difference is seen between testing groups (Fig. 2a, b). The animals were given 1 day of rest following surgery, before resuming Von Frey testing on consecutive days until they were euthanized on day-12 or day-18, depending on the testing group. Nanomedicine (CXB-NE) or vehicle (DF-NE) is injected on day-8 following surgery due to a significant increase (*p* < .0001) in pain-like hypersensitivity at this time-point in CCI compared to sham rats (Fig. 2a, b line 1). Following injection at day-8, nanomedicine treated rats (CXB-NE) show similar pain-like hypersensitivity to vehicle-treated rats (DF-NE), which is significantly higher (*p* < .0001) compared to sham rats (Fig. 2a, b line 2). At day-12 following surgery, CCI animals treated with nanomedicine (CXB-NE) showed a significant (*p* < .0001) reversal in pain-like hypersensitivity (Fig*.* 2a, b lines 4, 5); and the group that received vehicle treatment showed significantly increased (*p* < .0001) pain-like hypersensitivity (Fig. 2a, b line 3). At day-18 following surgery, the nanomedicine treatment group returned to levels of pain-like hypersensitivity resembling that of the vehicle group; a significant difference (*p* < .0001) from the pain-relief state seen at day-12 (Fig. 2a, b line 6).

### NIRF signal accumulation at the inflamed sciatic nerve of live animals is lowered after nanomedicine treatment

NIRF signal in live animals is imaged in a preclinical fluorescence imager under anesthesia before and after injection of nanomedicine (CXB-NE) or vehicle (DF-NE) on day-8 after surgery, and then on day-11 in one group, and day-17 in another. We have previously shown that nanoemulsion is phagocytosed by macrophages [29, 50], before infiltrating the sciatic nerve of CCI rats—and that a measurable NIRF signal is detected above the ipsilateral sciatic nerve in live animals at day-11 post-surgery [31, 63]. In the present study, we show that at pre-injection (day-8), there is no NIRF signal above the ipsilateral sciatic nerve of CCI animals (Fig. 2d). By day-11 we see a strong signal in the vehicle-treated CCI animals (Fig. 2e) and a significantly (*p* = .0173) reduced NIRF signal in nanomedicine treated animals (CXB-NE) (Fig. 2c, f). At day-17 post-surgery, there is no effect of nanomedicine treatment, however, the NIRF signal in both groups (Fig. 2g, h) resembles the level seen in the nanomedicine treated group at day-11. The NIRF signal in the day-17 vehicle-treated group (Fig. 2c, g) is significantly lower (*p* = .0040) than the day-11 vehicle group (Fig. 2c, e).

### Ex-vivo tissue analysis of macrophage infiltration at the affected sciatic nerve confirms a reduction in inflammation

**Fig. 3:**
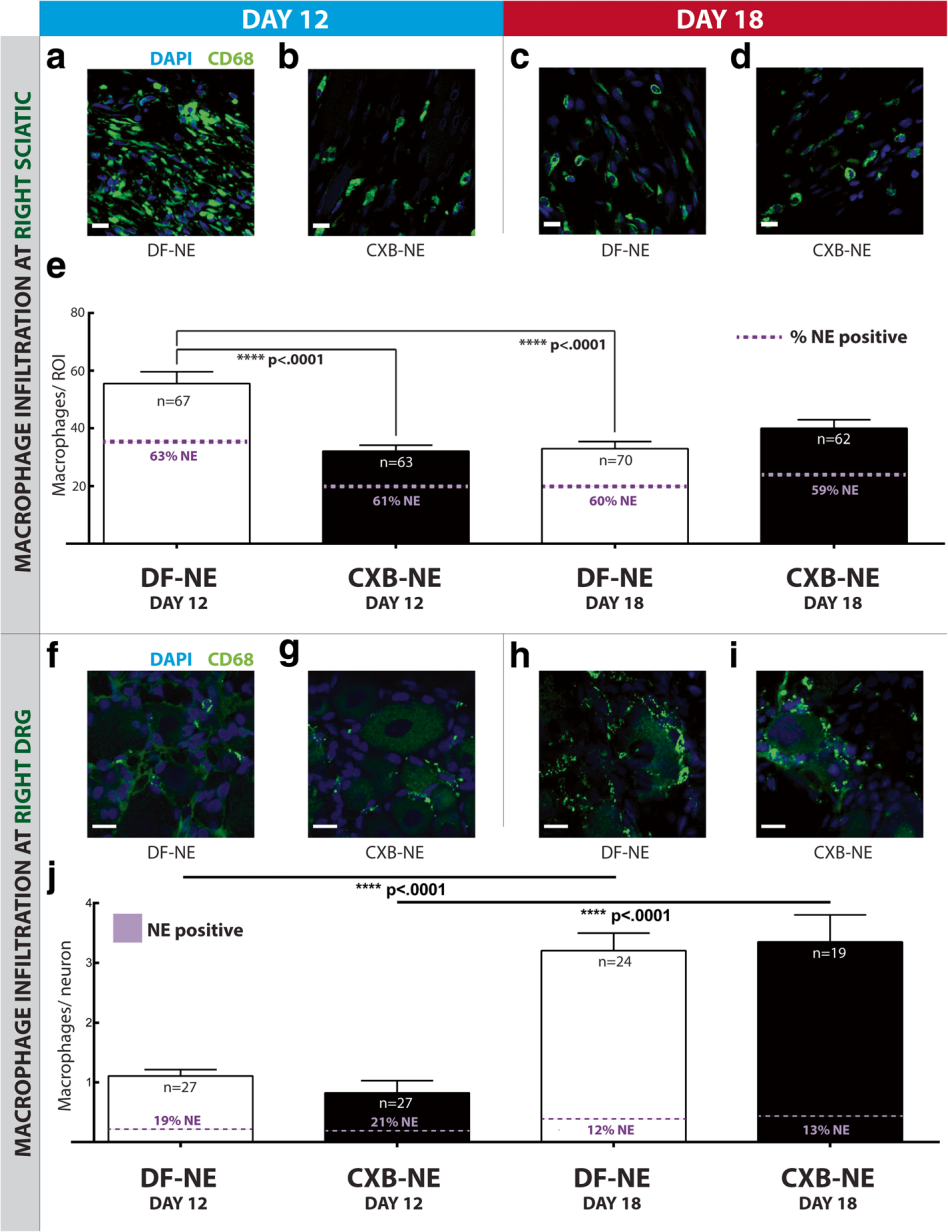
Macrophage infiltration at the ipsilateral sciatic nerve and associated DRG. At day-12, macrophage infiltration to the ipsilateral sciatic nerve is significantly reduced in the CXB-NE group (**b** and **e**), compared to the DF-NE group (**a** and **e**) (*p* < .0001). There is no significant difference between DF-NE and CXB-NE groups at day-18 (**c**, **d** and **e**). At both day-12 and day-18, the percentage of sciatic nerve infiltrating macrophages that are positive for nanomedicine ranges from 59 to 63% (**e**). A measure of macrophage infiltration per DRG cell body is calculated. These percentages are lower at the DRG: 19% in the day-12 DF-NE condition, 21% in the day-12 CXB-NE condition, 12% in the day-18 DF-NE condition and 13% in the day-18 CXB-NE condition (**j**). Seen here in this animation are nanoemulsion droplets inside macrophages [58]: 10.6084/m9.figshare.8142962. In the DRG, there is no significant difference between the DF-NE and CXB-NE groups at both day-12 and day-18. Macrophage infiltration at the ipsilateral L4 and L5 DRG is significantly higher at day-18 in both the DF-NE (*p* < .0001) and CXB-NE (*p* < .0001) groups (**h**, **i** and **j**) compared to respective groups at day-12 (**f** and **g**). All scale bars are 15 μm. Data is represented as mean ± SEM (*n* = 3 animals, 19–42 ROI; ****p < .0001, one-way ANOVA with Tukey’s post hoc test)

Macrophage infiltration at the ipsilateral sciatic nerve of CCI rats was assessed by anti-CD68 immunofluorescence staining. A significant reduction (*p* < .0001) in infiltration is revealed at day-12 in nanomedicine (CXB-NE) treated rats (Fig. 3b, e). At day-18, there is no effect of CXB-NE treatment (Fig. 3c, d), and significantly reduced (*p* < 0.0001) macrophage infiltration in the vehicle group (DF-NE) compared to day-12 (Fig. 3a, c). Infiltration in the day-18 nanomedicine treatment group resembles levels seen in the day-12 CXB-NE and day-18 DF-NE groups (Fig. 3b, c, d). The percentage of infiltrating macrophages that are positive for nanomedicine NIRF signal was analyzed, and in both conditions at the injured sciatic nerve, constitutes approximately 60% of macrophages (Fig. 3e).

### Nanomedicine treatment does not reduce macrophage infiltration at the L4 and L5 DRG associated with the inflamed sciatic nerve

The L4 and L5 DRG are associated with the sciatic nerve—approximately 98–99% of all sciatic nerve DRG cell bodies are located here [3]. Macrophage infiltration at the DRG was quantified by calculating the average number of macrophages per cell body. CD68 analysis of DRG neurons revealed a significant increase of macrophage infiltration at day-18 compared to day-12 in both the vehicle (*p* < .0001) and nanomedicine (*p* < .0001) treated groups (Fig. 3f-j). Nanomedicine treatment did not significantly reduce macrophage infiltration to the ipsilateral DRG of CCI rats in either the day-12 or day-18 groups. Approximately 20% of macrophages infiltrating the DRG at day-12 are positive for nanomedicine. This percentage approximately halves at day-18, indicating clearance of macrophages, and infiltration of new monocytes.

### COX-2 positive macrophages in the ipsilateral sciatic nerve are significantly reduced following nanomedicine treatment

**Fig. 4:**
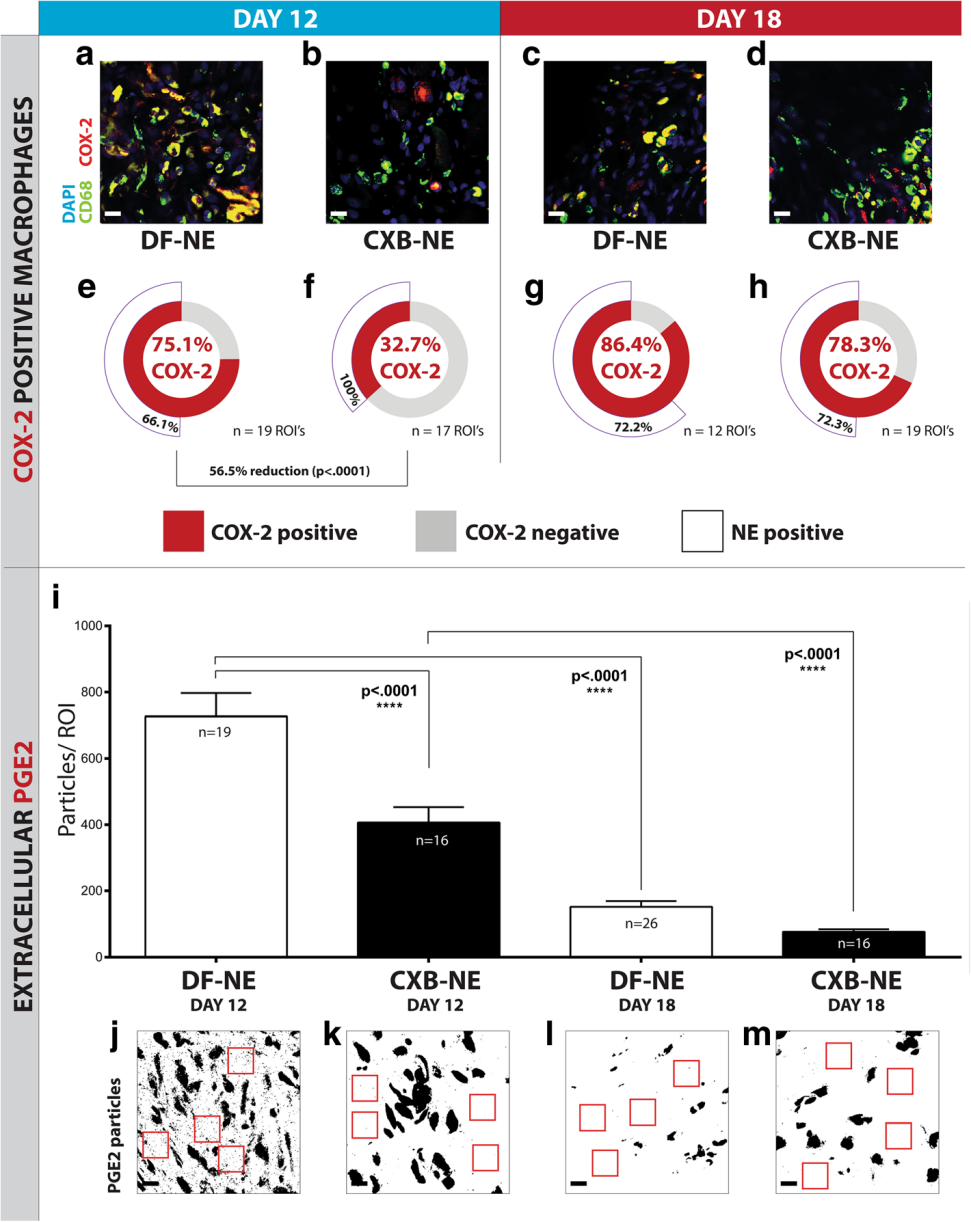
Macrophage COX-2 and extracellular PGE2 expression is reduced following CXB-NE treatment. Macrophage expression of COX-2 enzyme and extracellular expression of its synthesized cytokine, PGE2, is measured at the ipsilateral sciatic nerve. There is a significant 56.5% reduction (Fisher’s exact test, *p* < 0.0001) of COX-2 positive macrophages at day-12 in the CXB-NE group (**b** and **f**), compared to the DF-NE group (**a** and **e**). Extracellular PGE2 levels are also significantly reduced (*p* < 0.0001) (**i** and **k**) in the day-12 CXB-NE group (**k**) compared to the day-12 DF-NE group (**j**). The representative images**--j**, **k**, **l** and **m**—have been converted to binary images to more clearly reveal the extracellular PGE2, which is counted by applying a size threshold during analysis. The larger particles denote COX-2 stained macrophages, and the smaller particles represent extracellular PGE2, examples of which are represented within the red boxes. At day-18, the proportion of COX-2 positive macrophages in the DF-NE (**c** and **g**) and CXB-NE (**d** and **h**) groups rises to levels comparable to the day-12 DF-NE group (**e**). Extracellular PGE2 is significantly reduced at day-18 in both the DF-NE (**i** and **l**) (*p* < .0001) and CXB-NE (**i** and **m**) (*p* < .0001) groups. Macrophages positive for COX-2 were analyzed to report on their colocalization with nanomedicine NIRF signal (white segment in **e-f**). A significantly higher co-localization of nanomedicine with COX-2 positive macrophages was observed in the day-12 CXB-NE condition (*p* < .0001). All scale bars are 15 μm. The significance of COX-2 positive macrophage percent difference between conditions is represented as a Fisher’s exact test *p*-value; 95% confidence interval. Extracellular PGE2 data are represented as mean ± SEM (*n* = 3 animals, 16–26 ROI; ****p < .0001, one-way ANOVA with Tukey’s post hoc test)

Tissue sections from the ipsilateral sciatic nerve were multi-stained with anti-CD68 (macrophage marker) and an antibody against the COX-2 enzyme (Additional file 1: Table S1) as well as the nuclear stain DAPI. The fluorescence intensity from these stains, as well as the NIRF signal emitted from the nanomedicine, was captured with four laser imaging channels using confocal microscopy. By counting individually labeled macrophages during image analysis, in addition to a measurement of macrophage infiltration, it was possible to calculate the percentage of these infiltrating macrophages that were positive for COX-2 expression and the presence of nanomedicine. The dose of nanomedicine is not saturating; not every macrophage is expected to have accumulated nanomedicine. At day-12, a remarkable 56.5% reduction (Fisher’s exact test, *p* < .0001) in COX-2 positive macrophages was observed in rats that were treated with nanomedicine (CXB-NE) (Fig. 4a, b, e, f). Levels of COX-2 positive macrophages at day-18 were similar to those seen in the vehicle-treated day-12 group (Fig. 4a, c, d, e, g, h). Of the COX-2 positive macrophages, the highest extent of nanomedicine colocalization was seen in the day-12 nanomedicine treatment group (Fig. 4f)—significantly higher than all other groups (*p* < .0001). The attenuation of COX-2 by CXB-NE is believed to halt further induction of the enzyme. Protein staining for COX-2 is not indicative of whether the protein has been inactivated by celecoxib—although it is expected that it has been in the day-12 CXB-NE condition due to the reduction in PGE2.

### Extracellular PGE2 at the ipsilateral sciatic nerve is significantly reduced following nanomedicine treatment

The expression of extracellular PGE2 was measured as a particle count at the ipsilateral sciatic nerve of CCI rats. A significant reduction (*p* < .0001) of extracellular PGE2 was observed at day-12 in nanomedicine treated rats (Fig. 4i, j, k). This coincides with the observed reduction of macrophage-expressed COX-2 enzyme (Fig. 4b, f), from which PGE2 is synthesized. At day-18, extracellular PGE2 is significantly reduced: approximately 4-fold (*p* < .0001) in both the DF-NE group and CXB-NE group, compared to their respective groups at day-12.

### Nanomedicine treatment significantly reduces the number of M1 pro-inflammatory macrophages while increasing the number of M2 anti-inflammatory macrophages in the sciatic nerve

**Fig. 5:**
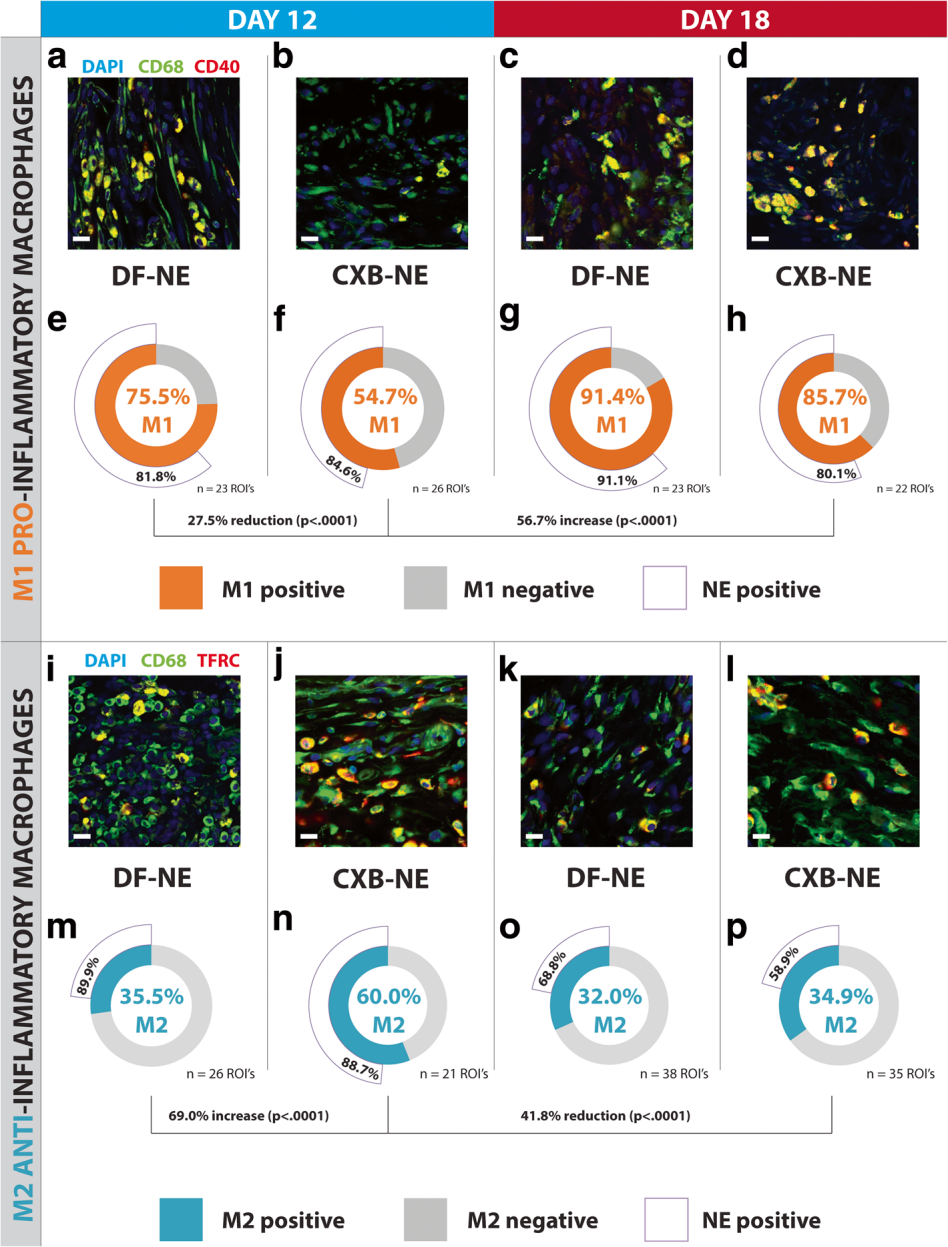
Macrophage polarity shifts from the pro-inflammatory M1 to anti-inflammatory M2 phenotype in the day-12 CXB-NE group. There is a 27.5% reduction (*p* < .0001) in M1 pro-inflammatory macrophages in the day-12 CXB-NE group (**b** and **f**) compared to the day-12 DF-NE group (**a** and **e**). At day 18 the percentage of M1 macrophages increases by 56.7 to 85.7% in the nanomedicine treated (CXB-NE) rats, compared to day 12 (**d** and **h**). At day-18, levels of M1-positive macrophages rise to 91.4% per ROI in the DF-NE group (**c** and **g**). There are no significant differences in nanomedicine co-localization with M1 macrophages. The percentage of anti-inflammatory M2 macrophages increases significantly (*p* <.0001) by 69.0% in the day-12 CXB-NE group (**j** and **n**) compared to the DF-NE animals (**i** and **m**). At day-18, the proportion of M2 macrophages in the CXB-NE group (**l** and **p**) drops significantly (*p* < 0.0001) by 41.8%, whilst there is no significant difference in the DF-NE group at day-18 (**k** and **o**), compared to day-12 (**i** and **m**). M2 macrophages in the day-18 conditions show a significantly lower nanomedicine NIRF colocalization compared to both the DF-NE (Fisher’s exact test, *p* < .0001) and CXB-NE (Fisher’s exact test, *p* < .0001) day-12 groups. At day 18, the percentage of M2 macrophages that are positive for nanomedicine NIRF signal is significantly lower in the CXB-NE group (Fisher’s exact test, *p* = .000376) compared to the DF-NE group. All scale bars are 15 μm. The significance of M1 and M2 positive macrophage percent difference between conditions is represented as a Fisher’s exact test *p*-value; 95% confidence interval. *n* = 3 animals, 21–33 ROI

Tissue sections from the ipsilateral sciatic nerve were stained with CD68 (macrophage marker), nuclear stain DAPI and an antibody against the CD40, a marker for M1 pro-inflammatory macrophages (Additional file 1: Table S1). In a separate experiment, anti-TFRC (Additional file 1: Table S1), a marker for M2 anti-inflammatory macrophages was co-stained alongside CD68-positive macrophages and DAPI. The fluorescence signal from these stains, as well as the NIRF signal emitted from the nanomedicine, was captured in multiple imaging channels using confocal microscopy. The percentage of macrophages positive for either M1 or M2 markers was calculated, in addition to counting the proportion of these cells positive for nanomedicine. A 27.5% reduction (Fisher’s exact test, *p* < .0001) of M1 macrophages was measured in ipsilateral sciatic nerve tissue of nanomedicine treated (CXB-NE) CCI rats (Fig. 5b, f) compared to the day-12 vehicle treatment group (Fig. 5a, e). At day 18, the percentage of M1-positive macrophages in the nanomedicine (CXB-NE) group increased by 56.7% (Fisher’s exact test, *p* < .0001), compared to day 12. There is a remarkably significant 69.0% increase (Fisher’s exact test, *p* < .0001) in the number of M2 anti-inflammatory macrophages found in nanomedicine treated rats at day-12, compared to the vehicle group Fig. 5i, j, m, n). The proportion of M2-positive macrophages shows a decrease of 41.8% (Fisher’s exact test, *p* < .0001) at day-18 compared to day-12 in nanomedicine treatment groups (Fig. 5j, n, l, p). Nanomedicine NIRF colocalization with M2 macrophages is significantly lower in both the DF-NE (Fisher’s exact test, *p* < .0001) and CXB-NE (Fisher’s exact test, *p* < .0001) groups at day 18 compared to day 12 (Fig. 5m-p), suggesting that there is a population of M2 macrophages that have fused to form MGCs. At day 18, nanomedicine NIRF colocalization with M2 macrophages is significantly lower (Fisher’s exact test, *p* = .000376) in the CXB-NE condition compared to the DF-NE condition, likely indicating that a greater proportion of M2 macrophages have fused to form MGCs.

### CD68-positive multinucleated giant cells in the ipsilateral sciatic nerve appear prominently by day-18 following surgery, and at significantly higher counts following nanomedicine treatment

**Fig. 6:**
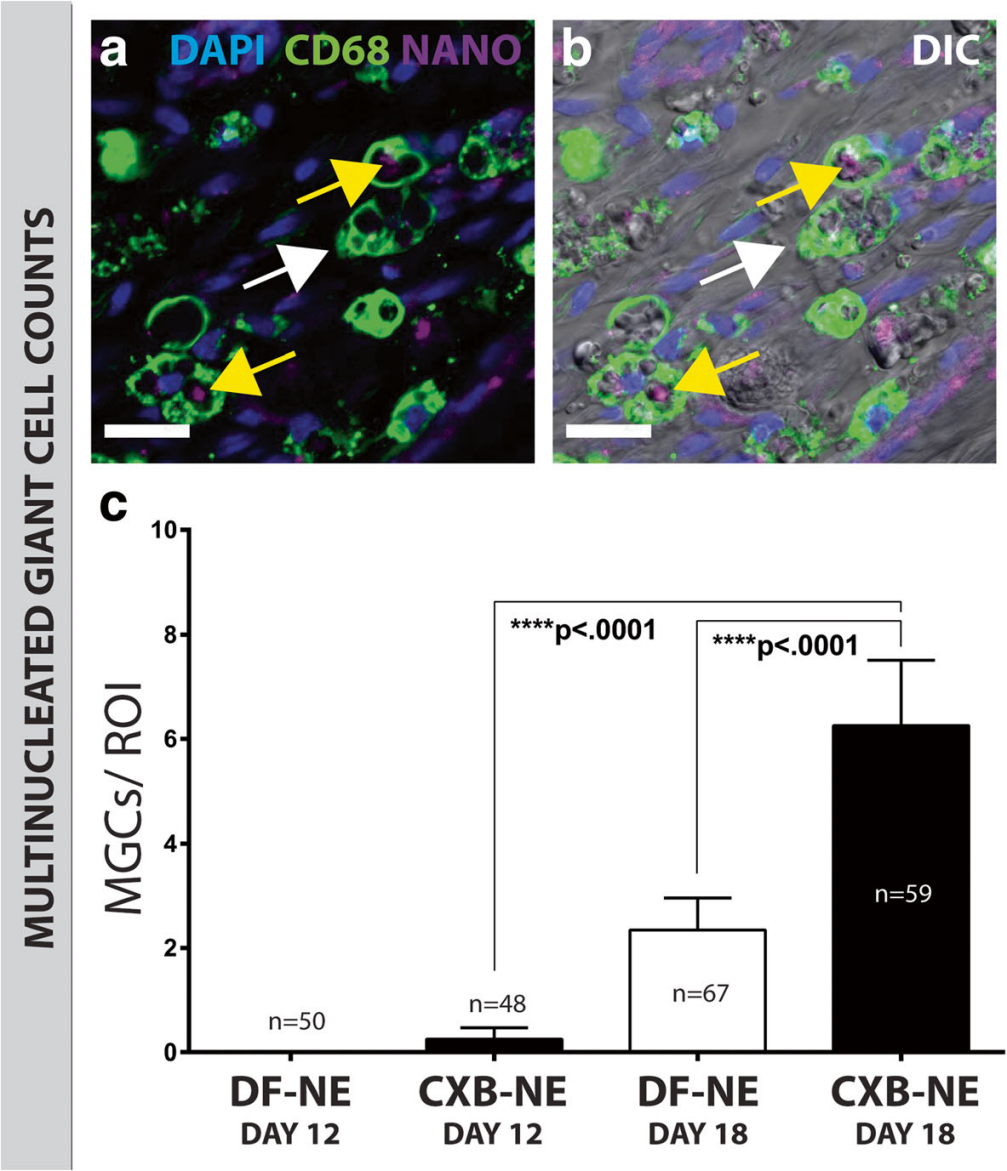
Macrophage fuse to form multinucleated giant cells at day-18. Macrophages can fuse to form multinucleated giant cells (**a**, **b**). MGCs were observed at higher numbers at day-18, in both the CXB-NE and DF-NE conditions (**c**). The day-18 CXB-NE group had significantly more MGCs compared to the day-18 DF-NE (*p* < .0001) and the day-12 CXB-NE (*p* < .0001) groups (**c**). White arrows point to CD68 staining showing macrophages fused into MGCs. Yellow arrows indicate a nanomedicine signal inside MGCs. Seen here in this animation are M2 macrophages fused to form a multinucleated giant cell [59]: 10.6084/m9.figshare.8142950. DAPI stained nuclei are blue, CD68 stained MGCs composed of macrophages are green, and the NIRF from nanomedicine is purple. Panel **a** is a merge of DAPI, CD68 and nanomedicine channels; and panel **b** is a merge that additionally includes a DIC channel in order to visualize nerve tissue morphology. All scale bars are 20 μm. Data are represented as mean ± SEM (*n* = 3 animals, 48–59 ROI; ****p < .0001, one-way ANOVA with Tukey’s post hoc test)

During an inflammatory reaction, monocytes and macrophages can fuse to form multinucleated giant cells (Fig. 6a, b). Composed of several fused cells, MGCs were observed to be approximately 20–30 μm at their greatest diameter (Fig. 6a, b; white arrows), and internalized NIRF signal from the nanomedicine was observed (Fig. 6a, b; green arrows). Positively stained with anti-CD68, virtually no MGCs were seen at day-12 in either the nanomedicine or vehicle treatment groups (Fig. 6c). However, MGCs were observed in greater quantities at day-18, with significantly higher counts (*p* < .0001) in the nanomedicine treatment group (Fig. 6c).

### The number of infiltrating mast cells in the ipsilateral sciatic nerve is significantly reduced following nanomedicine treatment and not in the ipsilateral DRG

**Fig. 7:**
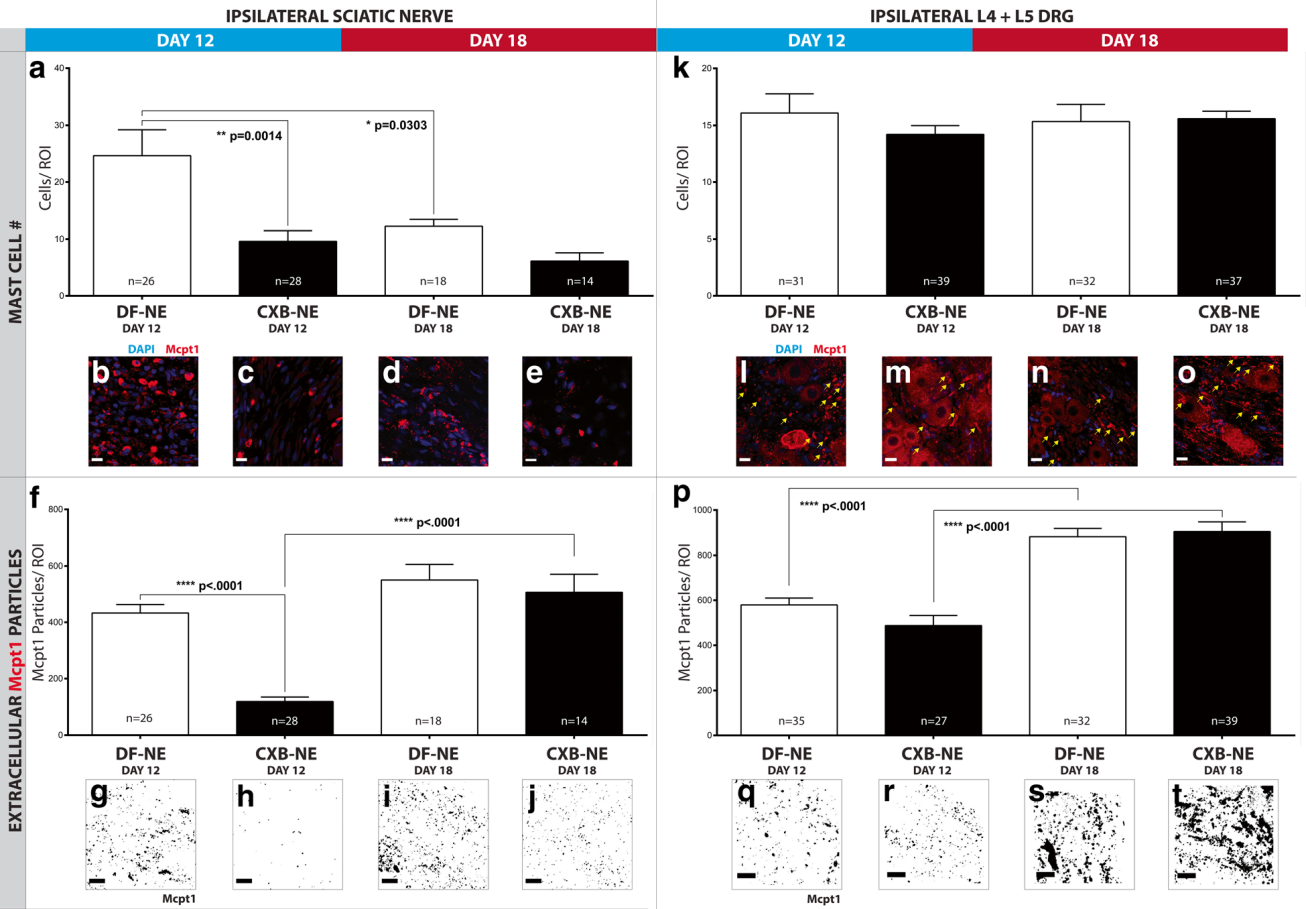
Mast cell number and extracellular Mcpt1 particles are lowered in ipsilateral sciatic nerve following CXB-NE treatment. The number of mast cells and extracellular Mcpt1 particles, both stained with anti-Mcpt1 antibody, are counted for each region of interest on tissue sections at the ipsilateral sciatic nerve—as well as ipsilateral L4 and L5 DRG—of CCI animals at both day-12 and day-18. At the ipsilateral sciatic nerve, mast cell number per ROI decreases significantly (**a**) in the CXB-NE condition (**a** and **c**) at day-12 (*p* = .0014) compared to the DF-NE condition (**a** and **b**). At day 18, both the CXB-NE (**a** and **e**) and DF-NE (**a** and **d**) conditions have significantly fewer mast cells per ROI compared to the DF-NE condition at day-12. Also notable is that there are significantly less (*p* = .0303) mast cells per ROI at day-18 (**a** and **d**) in the DF-NE condition compared to day-12 (**a** and **b**). There are no significant differences in mast cell counts among treatment groups in the DRG (**k**) (mast cells are indicated by yellow arrows in **l**, **m**, **n,** and **o**). The representative images**—g**, **h**, **i** and **j**—have been converted to a binary format to better visualize Mcpt1 particles, which are counted by applying a size threshold during analysis. Mcpt1 particles per ROI are significantly reduced (*p* < .0001) at the ipsilateral sciatic nerve milieu in the day-12 CXB-NE condition (**f** and **h**) compared to the day-12 DF-NE group (**f** and **g**). Interestingly, the particle number per ROI remains at similar levels in the day-18 DF-NE group (**f** and **i**) and is observed at similar levels again in the day-18 CXB-NE group (**f** and **j**), a significant increase (*p* < .0001) compared to the day-12 CXB-NE group (**h**). In the DRG, there is no significant difference between treatment conditions in either the day-12 or day-18 groups (**p**, **q**, **r**, **s**, and **t**), however, both conditions show a significant increase in Mcpt1 particles at day-18 (*p* < .0001). All scale bars are 15 μm. All images were resolved with a Nikon 40x oil immersion objective. Data is represented as mean ± SEM (*n* = 3 animals, 14–26 ROI; *p < .05, **p < .01, ****p < .0001, one-way ANOVA with Tukey’s post hoc test)

Mast cells are a key component of the inflammatory response and are integral in the potentiation of chronic pain [10]. In addition to resident populations in most tissues, there is a circulation of mast cell progenitors in the blood, which can infiltrate sites of inflammation [14]. Given the crosstalk between mast cells, other immune cells—such as macrophages—and the nervous system, we investigate mast cell expression at the ipsilateral sciatic nerve and associated L4 and L5 DRG. The number of mast cells – indicated by positive staining for Mcpt1—were counted for each region of interest from sciatic nerve tissue and DRG sections. The number of mast cells per ROI was significantly reduced (*p* = .0014) in the CXB-NE nanomedicine treated rats (Fig. 7c) at day-12 compared to the DF-NE vehicle-treated group (Fig. 7b). There was a reduction in mast cell numbers in both treatment groups at day-18 (Fig. 7d, e). There were significantly less (*p* = .0303) mast cells per ROI at day-18 in the vehicle treatment group (Fig. 7d) as compared to day-12 (Fig. 7b). In the location of the ipsilateral DRG cell bodies, there are no significant differences between mast cell numbers among treatment groups, suggesting that the cells are resident in this location, and have not infiltrated.

### Mast cell degranulation is significantly reduced following nanomedicine treatment

The diverse effector functions of mast cells are mediated by the secretion of a wide variety of biologically active products that are contained in secretory granules. Extracellular staining of Mcpt1 was indicative of a major component of mast cell granules, and particle counts were interpreted as the extent of mast cell degranulation. The number of Mcpt1 particles per ROI was significantly reduced (*p* < .0001) in the ipsilateral sciatic nerve milieu in the CXB-NE nanomedicine treated rats at day-12 (Fig. 7f, h) compared to the day-12 vehicle-treated group (Fig. 7f, g). It was observed that the granule number per ROI remains at similar levels in the day-18 vehicle group (Fig. 7f, i) and is measured at similar levels again in the day-18 nanomedicine treated group (Fig. 7f, j), a significant increase (*p* < .0001) compared to the day-12 nanomedicine treated rats (Fig. 7f, h). In the ipsilateral DRG, there is no treatment effect influencing extracellular Mcpt1 particles (Fig. 7k), however, there is a significant increase (Fig. 7p, s, t) in both treatment conditions at day-18 compared to day-12 (*p* < .0001).

## Discussion

Here we report that a single low dose of celecoxib delivered to macrophages via nanomedicine leads to a reduction in hypersensitive pain-like behavior, persisting for approximately 6 days. During this state of pain relief, our data reveals that the number of infiltrating macrophages at the site of chronic constriction injury is reduced in the drug-treated condition. We also show a significant reduction in both COX-2 positive macrophages and extracellular PGE2 in the milieu of the nerve injury, when the drug is present. Furthermore, the drug influences a shift in the population of macrophages to the M2 anti-inflammatory state. Finally, we observe that the presence of the drug reduces mast cell activation at the site of injury, indicated by a lower number of mast cells and extracellular Mcpt1—indicative of secreted granules. Our study was designed to investigate the inflammatory neuropathology framed around two time-points—the first, when CXB-NE rats experienced peak relief of neuropathic pain (day-12) and the second, when pain-like behavior had returned to levels similar to DF-NE vehicle-treated rats (day-18).

### Theranostic nanomedicine offers multi-day neuropathic pain relief, effectively diagnoses inflammation in-vivo and sheds light on the underlying mechanisms of immune cell pathology

Administering a single low dose of celecoxib (~0.24 mg/kg) to CCI rats via intravenous nanomedicine delivery is effective in reversing pain-like hypersensitivity as reported here and previously [31]. It is well known that NSAIDs like celecoxib have poor efficacy in treating neuropathic pain [42], however this is in the context of non-targeted approaches whereby drugs are delivered either orally or parenterally, and consequently made systemically available. Intrathecal injections also do not meet our design criteria as presented here. In our earlier work and in this study, we demonstrated a dramatic improvement in the efficacy of celecoxib. The key is that in this paradigm, the COX-2 inhibitor, celecoxib, is directly delivered to the target—the COX-2 enzyme in the monocyte, rather than other tissues. The nanoemulsion droplets provide a long-term intracellular depo for the drug to successfully inhibit the COX-2 enzyme in these cells. Dr. Janjic has designed this approach as a means of dramatically enhancing the efficacy of COX-2 inhibition in inflammatory diseases such as neuropathic pain. In earlier studies we have demonstrated that targeted COX-2 inhibition by nanoemulsions can produce marked pain relief in a rat model of neuropathic pain [31, 47]. This, in our view, promises to be both a safer and more effective strategy than CNS-targeted treatments such as opioids.

This study demonstrates a peak relief from pain on the third and fourth day after nanomedicine treatment and persists for up to 6 days after the injection (Fig. 2a, b). In addition to the therapeutic utility of the nanomedicine, it exhibits a diagnostic function—able to report on the amount of macrophage infiltration—an indication of the extent of underlying inflammation. The NIRF signal emitted by the nanomedicine is predictably reduced in nanomedicine-treated rats when their right thighs are imaged at day-11 after surgery (Fig. 2c, f). However, when imaged the night before being euthanized at day-18 post-surgery, the NIRF signal is reduced in both the nanomedicine and vehicle-treated groups (Fig. 2c, g, h). This may be due to the turnover of macrophages and the relative clearance of the nanomedicine over time from the body of the rats. We have shown in a biodistribution study that the relative NIRF signal emitted from the liver in both nanomedicine and vehicle-treated rats is reduced at day-18 as compared to day-12 after surgery (Additional file 1: Figure S1).

### Macrophage infiltration is reduced in the inflamed sciatic nerve and not the associated L4 and L5 DRG following nanomedicine treatment

An inflammatory response that is localized to the nervous system is termed neuroinflammatory and can be caused by infection, autoimmunity and tissue injury. These insults provide cues that are followed by the initiation of inflammation, which causes plasma extravasation and infiltration of immune cells such as neutrophils, T cells, and monocytes, as well mobilizing resident macrophages and mast cells [33]. Monocytes and neutrophils are circulating phagocytes that can be signaled during an immune response. Monocytes are especially dynamic because they can be signaled from the blood circulation to infiltrate sites of inflammation and differentiate into macrophages. They are also the most abundant infiltrating immune cell arriving at injured nerve tissue [37]. This blood to tissue migration of the monocyte—as well as its phagocytosis of foreign materials—underpins the design of nanomedicine targeting the inflammation that gives rise to chronic pain. The hypothesis: to attenuate COX-2 activity in these monocytes fated to infiltrate the CCI sciatic nerve using nanomedicine loaded with celecoxib. Reducing COX-2, in turn, reduces the recruitment of additional immune cells and, hence reduces inflammation.

We have previously shown that fewer macrophages infiltrate the injured sciatic nerve at day-12 following nanomedicine treatment [31]. This study sought to report on the extent of infiltration at day-18—a time when pain relief has diminished. Additionally, we aimed to investigate the infiltration of monocytes into the associated L4 and L5 DRG of the sciatic nerve.

We confirm our previous finding that nanomedicine (CXB-NE) treated rats show a reduction in macrophage infiltration at the injured sciatic nerve tissue isolated at day-12 (Fig. 3). There is no effect of treatment at day-18, and additionally, there is no increase in infiltration at day-18; in fact, there is a significant reduction (*p* < .0001) in the vehicle-treated group (Fig. 3). It is anticipated that macrophage infiltration does not proceed at a constant rate throughout the inflammatory response and that by day-18, has shifted to a lower turnover rate—a point at which the macrophage population is contributing to both Wallerian degeneration and axonal regeneration [11].

The ipsilateral L4 and L5 DRG neurons associated with the injured sciatic nerve show no reduction in macrophage infiltration following nanomedicine treatment. As reported in a previous study [32], we show that macrophages accumulate over time in the chronic pain-affected DRG—with a significant increase in infiltration evident at day-18 compared to day-12.

The rationale for focusing the present study on monocyte phagocytosis of nanomedicine and migration to the injured sciatic nerve is due to this cell type being the most abundant [37] at sites of tissue injury. Neutrophils are another type of phagocyte that are abundant early at the site of injury or infection [2], however our earlier reports confirm specificity for monocyte uptake of the designed nanomedicine, and the selective inhibition of COX-2 intracellularly using this approach [47]. Numerous studies have also shown preference of nanoemulsion uptake into monocytes compared to other cell types [24, 45, 62]. Interestingly, it has recently been shown that neutrophils are not a phenotypically homogenous population as originally believed, and that like macrophages, possess a phenotypic versatility based on function [56]. It would hence be prudent in the future to design new nanoemulsions specifically targeted to neutrophils in order to study their phagocytic and transport potential in the context of neuroinflammation and pain pathology in the PNS.

### Nanomedicine treatment significantly reduces the proportion of COX-2 positive macrophages and extracellular PGE2 at day-12 but not day-18

In the day-12 nanomedicine treatment group, we demonstrate a significant reduction (*p* < .0001) in the percentage of COX-2 positive macrophages (Fig. 4), and the quantity (*p* = .0029) of extracellular PGE2 (Fig. 4) at the injured sciatic nerve. The percentage of COX-2 positive macrophages at day-18 reverts to a level resembling that in the day-12 vehicle-treated group, whilst extracellular PGE2 levels fall significantly (*p* < .0001) in both treatment groups at day-18 (Fig. 4). We focus here on macrophages either being positive or negative for COX-2 and subsequently reporting a percentage that is positive. In terms of PGE2, we shift the focus on extracellular expression; the rationale being that a release of PGE2 from macrophages is a more conclusive measure of a pro-inflammatory state. Additionally, cells that are positive for both COX-2 and nanomedicine are revealed; there is a higher percentage of nanomedicine-positive macrophages (also positive for COX-2) in the day-12 nanomedicine treated group, compared to all other groups. The celecoxib treatment attenuates COX-2 by blocking the protein’s activity [14], not destroying COX-2.

### Nanomedicine treatment drives macrophages to switch polarity to an anti-inflammatory phenotype

The macrophage is a pleiotropic cell type, that not only promotes inflammation but is also involved in its resolution, as well as tissue repair and remodeling [16, 36, 40]. A heterogeneous population of macrophages can switch phenotype to serve these diverse functions; they can acquire pro-inflammatory (termed M1), and anti-inflammatory (termed M2) phenotypes. It was a goal of this study to determine the pro-inflammatory and anti-inflammatory phenotypes of infiltrating macrophages to the site of sciatic nerve injury, and subsequently, determine if the composition of M1 and M2 macrophages changes in response to nanomedicine treatment. A recent proteomic study [5] that proposed cell membrane markers to precisely discriminate M1 and M2 macrophages was consulted in order to co-stain macrophages with an M1 or an M2 marker. The antibody against TNF receptor superfamily member 5 (anti-CD40) is a marker for M1 macrophages, and the antibody against the transferrin receptor (anti-TFRC) is a marker for M2 macrophages [5].

At day-12, there is a significant decrease (*p* < .0001) in the percentage of macrophages positive for the M1 marker following CXB-NE nanomedicine treatment (Fig. 5). Remarkably, the reduction in M1 macrophage percentage persists at day-18 in the nanomedicine group (Fig. 5). In an experiment investigating M2 macrophages at the injured sciatic nerve, nanomedicine treatment resulted in a significantly higher (*p* < .0001) percentage of the M2 phenotype at day-12 compared to the vehicle group (Fig. 5). At day-18, the percentage of M2 macrophages in the nanomedicine treatment group decreases significantly (*p* < .0001), compared to day-12 (Fig. 5). The increase in M2 macrophages appears to be lower than the reduction in M1 macrophages and could be explained by the fact that the M2 phenotype is the default state of resident macrophages [28], hence why the differences in the M2 phenotype proportion are not as pronounced as M1.

This result indicates an important shift in macrophage polarity from M1 to M2 in the pool of macrophages at the injured sciatic nerve following nanomedicine treatment. Taken with our findings regarding macrophage COX-2 and external PGE2 expression, the polarity shift indicates that the nanomedicine is attenuating COX-2 activity inside the macrophage, leading to a reduction in extracellular PGE2 and by doing so is switching the macrophage to an anti-inflammatory M2 phenotype. This M2 phenotype is consequently equipped to promote axonal regeneration [11].

### Multinucleated giant cells form from M2 macrophages and are observed at day-18, predominantly in the nanomedicine treatment group

We noted the presence of significantly more multinucleated giant cells in the nanomedicine treated rats at day-18. These are formed from the fusion of their M2 polarized macrophage precursors [27, 51], and function to more effectively phagocytose relatively large debris from tissues. Significantly more MGCs are observed in the nanomedicine treated group at day-18. Building on the finding that there is a polarity shift towards the M2 phenotype, it can be hypothesized that M2 macrophages have fused into MGCs in order to perform their tissue healing functions in a more efficient manner. This is further evidenced by comparing the nanomedicine NIRF colocalization with M2 macrophages between day-12 and day-18 groups. There is a significant decrease in nanomedicine-positive M2 macrophages at day-18 in both the DF-NE (Fisher’s exact test, *p* < .0001) and CXB-NE (Fisher’s exact test, *p* < .0001) groups. Additionally, the increase in MGCs in the CXB-NE condition at day-18 coincides with a decrease in nanomedicine NIRF positive M2 macrophages (Fisher’s exact test, *p* = .000376); this suggests that more M2 macrophages have fused to form MGCs in this condition.

### The shift in macrophage polarity is associated with a reduction in mast cell activation

**Fig. 8:**
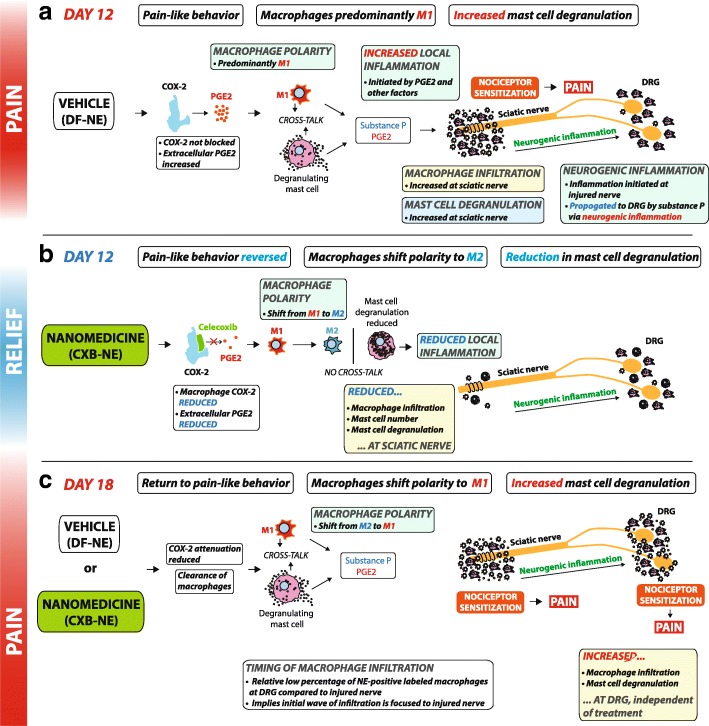
Proposed mechanisms underlying reversal of pain-like behavior at day-12 and diminished relief at day-18. In a neuropathic pain state, CCI rats administered with DF-NE vehicle nanomedicine exhibit pain-like behavior (**a**). This is proposed to be centrally driven by the attenuation of COX-2 by celecoxib delivered to circulating monocytes by nanomedicine. The polarity of macrophages at the injured sciatic nerve is predominantly the M1 pro-inflammatory phenotype. This influences crosstalk with mast cells, and their subsequent activation. With both immune effector cell types in an activated state, local inflammation is increased, resulting in a subsequent increase in macrophage infiltration. Inflammation at the ipsilateral DRG is transmitted through the afferent nociceptors from the injured sciatic nerve via neurogenic inflammation. In nanomedicine treated rats in the day-12 group (**b**), there is a reduction in COX-2 positive macrophages and extracellular PGE2 via the action of nanomedicine-delivered celecoxib. There is both a reduction in macrophage infiltration to the injured sciatic nerve and a shift of macrophage phenotype from pro-inflammatory (M1) to anti-inflammatory (M2). There is no nanomedicine treatment effect of macrophage infiltration to the DRG at day-12. M2 macrophages function to repair and regenerate the injured tissue and do not crosstalk with resident mast cells. There is a return to pain-like behavior at day-18 (**c**) in both nanomedicine and vehicle-treated groups. Macrophage infiltration to the injured sciatic nerve at day-18 is relatively low in both treatment groups—similar to levels observed in the day-12 nanomedicine-treated group. Macrophage infiltration to the DRG is increased at day-18, compared to day-12 and there are no significant differences between treatment groups. It is proposed that inflammation is initiated at the injured nerve and propagated to the associated L4 and L5 DRG via neurogenic inflammation. This drives recruitment of macrophages—as well as influencing further mast cell degranulation—and together provides the inflammatory input to sensitize nociceptors, resulting in an increase in pain-like behavior. The relatively low percentage of nanomedicine -positive macrophages at the DRG compared to the injured nerve suggests that the initial wave of macrophage infiltration is focused to the injured nerve

Mast cells mature from recruited progenitors released from the bone marrow into the blood circulation [14] and are a key effector cell of the innate immune system. They are involved in the first response to an insult to organs or tissues [53] and influence subsequent inflammatory events—for example, by activating macrophages [12, 55]. Factors released when mast cells degranulate can also sensitize nociceptors and lead to increased pain pathogenesis [1]. These can include TNAα [72], IL-1β [71], and tryptase—which interacts with protease-activated receptor 2 (PAR2) on nociceptors [38, 66]. This response manifests as the initiation, amplification, and prolonging of inflammation. We report here that the number of mast cells is significantly decreased (*p* = .0014) at day-12 in nanomedicine treated rats (Fig. 7). The mast cell number per ROI is not affected by drug treatment at day-18 (10 days after the single injection of nanomedicine). However, the vehicle-treated group reveals significantly fewer (*p* = 0.0303) mast cells at the injured sciatic nerve compared to day-12 (Fig. 7). Mast cell degranulation in the injured sciatic nerve is significantly lowered at day-12 in the CXB-NE nanomedicine treated rats, indicated by a reduction in extracellular Mcpt1 particles. By day-18, levels of Mcpt1 particles in the nanomedicine treated rats revert to levels resembling the vehicle treatment group at day-12, increasing significantly (*p* < .00001). In the DRG, there is no treatment effect either at day-12, or day-18, which could suggest that the mast cells in this location are resident and have not infiltrated from blood-borne progenitors. There is, however, a significant increase (*p* < .0001) in mast cell degranulation in ipsilateral DRG in both treatment conditions at day-18, compared to day-12. Much like the increase in macrophage infiltration to the DRG observed at day-18, it is proposed that the increased mast cell degranulation is a result of ongoing neurogenic inflammation conducted from the injured sciatic nerve (Fig. 8).

Based on our data, we have proposed mechanisms for the immune-cell neuropathology underlying the neuropathic pain state, a state where there is pain relief and a final state where there is a return to pain-like behavior (Fig. 8). Taken together, our results suggest a link between the predominant pool of M1 macrophages at the injured sciatic nerve of rats administered with DF-NE vehicle nanomedicine, along with a higher percentage of COX-2 positive macrophages and a higher expression of extracellular PGE2. This inflammatory macrophage phenotype functions as an immune effector system, signaling to other cells—such as resident mast cells—to perpetuate inflammation via their activation, and subsequent degranulation (Fig. 8a). The majority of M1 macrophages are fated to die, terminated by their nitrous oxide production [28], whilst M2 macrophages are involved in resident tissue functions such as repair and regeneration, which conceivably requires them to be alive for longer.

We propose that in the ‘pain relief’ state driven by CXB-NE nanomedicine, the inactivation of COX-2 and reduction in the production and release of PGE2 causes a shift towards an anti-inflammatory M2 macrophage phenotype (Fig. 8b). There is hence a reduction in macrophage crosstalk with mast cells—i.e. they are not activated—resulting in reduced local inflammation at the ipsilateral sciatic nerve. An effect of reduced inflammation is a lower potential for macrophage and mast cell infiltration, a result which we observe.

### The proposed mechanism underlying the return to pain-like behavior at day-18

Our data indicate that the increase in macrophage infiltration to the L4 and L5 DRG associated with the injured nerve of CCI animals, as well as the increase in mast cell degranulation, may be driving the return to pain-like behavior observed at day-18 (Fig. 8c). It has been demonstrated that the tactile allodynia underpinning pain-like behavior is dependent on peripheral macrophages [13]. In addition to the immune cell infiltration to the site of nerve injury, it has been shown that macrophages are also abundant at the DRG [26, 41], and are observed to circle the cell bodies of injured A-fiber sensory neurons [65]. By observing the NIRF labeling of macrophages with nanomedicine, our data give us clues as to the relative timing of macrophage infiltration to the injured sciatic nerve and its associated DRG. It was observed that a lower percentage of macrophages infiltrating the DRG were nanomedicine positive—in both treatment groups—compared to the sciatic nerve, which suggests that the initial wave of infiltration is focused to the site of CCI surgery. It is thought that this initial wave of immune cell migration is initiating inflammation at the sciatic nerve and sensitizing its nociceptors. It is possible that the neuroinflammatory state at the DRG—characterized by the increase in macrophage infiltration—is propagated from this sciatic nerve sensitization via a process of neurogenic inflammation [70]. Substance P, a neuropeptide that perpetuates the conduction of neurogenic inflammation, is released by both macrophages and mast cells and acts on peripheral nociceptors [10]. Inflammation is consequently perpetuated to the DRG from the peripheral nerve. Additionally, we have previously shown an elevated DRG expression of the TRPV1 receptor central in pain transmission that is also labelled with a retrograde dye applied to the footpad [63], confirming the path of neurogenic inflammation.

## Conclusions

Our results suggest that the central driver of nanomedicine chronic pain relief is a shift towards an M2 macrophage phenotype, via attenuation of intracellular macrophage COX-2. M2 macrophages at the injured sciatic nerve fuse to form MGCs, that tackle the phagocytosis of large debris; namely dead distal nerve fibers via Wallerian degeneration. This population of anti-inflammatory macrophages is also contributing to axonal nerve regeneration. The shifting towards an anti-inflammatory milieu at the injured nerve is thought to result in less M1 macrophage recruitment—reducing inflammation, and subsequent neuropathic pain. In the absence of the CXB-NE COX-2 targeted nanomedicine, it is posited that the resulting pro-inflammatory environment at the injured nerve consists in part of M1 macrophages signaling the upregulation and activation of mast cells—further perpetuating neuroinflammation, and contributing to a propagation towards the CNS, via neurogenic inflammation. Our data also points to a possible mechanism underlying the return to pain-like behavior--it becomes clear that the locale of the associated DRG is not influenced significantly by the day-8 macrophage-targeted treatment. This suggests that in the absence of therapeutic influence, the neuroinflammatory milieu of increasing macrophage infiltration to the DRG, and increased mast cell degranulation is sensitizing nociceptors, causing a return to neuropathic pain. Taken together, this report suggests for the first time that a pain nanomedicine phagocytosed by circulating monocytes that infiltrate the site of injury shifts their polarity via COX-2 attenuation, reduced PGE2 synthesis, and in turn, influence a reduction in mast cell activation—resulting in multi-day neuropathic pain relief.

Moreover, our investigation suggests that while immune neuropathological events at the site of the injured nerve can be successfully reversed with targeted immune-cell therapy, the spatial signature of neuroinflammation towards the CNS must also be concurrently addressed. The utility of a nanomedicine targeted to immune cell pathology offers a new research paradigm that can yield dynamic investigation and tracking of temporal patterns of cell infiltration, phenotypic change, and alterations in gene expression.

## Additional file

Additional file 1: **Table S1.** Primary antibodies used for immunofluorescence. **Table S2.** Secondary antibodies used for immunofluorescence. **Figure S1.** Clearance of NIRF signal from the liver. (PDF 307 kb)

## Data Availability

The datasets generated and/or analyzed during the current study are available in the Figshare repository.

## Abbreviations

CCI: Chronic constriction injury
COX-2: Cyclooxygenase-2
CXB-NE: Celecoxib nanoemulsion
DF-NE: Drug-free nanoemulsion
DRG: Dorsal root ganglia
MGC: Multinucleated giant cell
NE: Nanoemulsion
NIR: Near-infrared
NIRF: Near-infrared fluorescent
NSAID: Nonsteroidal anti-inflammatory drug
PGE2: Prostaglandin E2
TFRC: Transferrin receptor
