# Supplementary material for: Nanomedicine-driven neuropathic pain relief in a rat model is associated with macrophage polarity and mast cell activation
Source: Acta Neuropathol Commun. 2019 Jul 5;7:108. doi: 10.1186/s40478-019-0762-y (PMC6612172; doi:10.1186/s40478-019-0762-y)
Supplement: Supplementary file 1 — Table S1. Primary antibodies used for immunofluorescence. Table S2. Secondary antibodies used for immunofluorescence. Figure S1. Clearance of NIRF signal from the liver. (PDF 307 kb) [file 40478_2019_762_MOESM1_ESM.pdf]

Nanomedicine-driven neuropathic pain relief in a rat model is associated with macrophage polarity and mast cell activation

Acta Neuropathalogica Communications

Muzamil Saleem [1,3], Brooke Deal [1,3], Emily Nehl [4], Jelena M Janjic [2,3], John A Pollock [1,3]

1. Department of Biological Sciences, Duquesne University, Pittsburgh, PA, USA
2. Graduate School of Pharmacy, Duquesne University, Pittsburgh, PA, USA
3. Chronic Pain Research Consortium, Duquesne University, Pittsburgh, PA, USA
4. Jacobs School of Medicine and Biomedical Sciences, University of Buffalo, Buffalo, NY, USA

Supplementary materials and methods

Ex-vivo imaging of whole liver

The NIRF signal in whole dissected liver from rats euthanized at day-12 and day-18 was imaged with a preclinical fluorescence imager (LiCOR® Pearl Impulse from LI-COR Biosciences, Lincoln, NE). The NIR dye in the nanomedicine that has accumulated in labeled monocytes/ tissue macrophages fluoresces in the scanner and reveals biodistribution of the agent. Images in the fluorescent channel (785 nm excitation for 820 nm emission) and a white light channel are acquired and merged in the LiCOR Pearl Impulse Software (version 2.0) with linked look-up-tables (LUT) [1]. NIRF images were analyzed using Image Studio Lite Software (LI-COR Biosciences, Lincoln, NE) as previously described [1,2]. Briefly, a region of interest (ROI) was selected over the liver organ and total fluorescence was measured.

Supplementary tables

Supplementary Table 1. Primary antibodies used for immunofluorescence

Antibody	Source	Manufacturer	Cat. #	Dilution	Target
Anti-CD68	Mouse anti-Rat	BIO-RAD	MCA341R	1/100	Monocyte/ macrophage
Anti-CD68	Rabbit anti-Rat	Abcam	ab125212	1/100	Monocyte/ macrophage
Anti-COX2	Rabbit anti-Rat	Abcam	ab15191	1/100	COX-2 enzyme
Anti-PGE2	Rabbit anti-Rat	Bioss	bs-2639R	1/100	PGE2
Anti-CD40	Armenian Hamster anti-Rat	ThermoFisher Scientific	14-0402-82	1/500	CD40
Anti-TFRC	Mouse anti-Rat	ThermoFisher Scientific	13-6800	1/250	Transferrin receptor
Anti-Mcpt1	Goat anti-Rat	ThermoFisher Scientific	sc-17041	1/100	Mast Cell Protease 1 (C-15)

Supplementary Table 2. Secondary antibodies used for immunofluorescence.

Fluorophore	Source	Manufacturer	Cat. #	Dilution
Alexa Fluor 488	Donkey anti-Mouse	ThermoFisher Scientific	A-21202	1/200
Alexa Fluor 488	Chicken anti-Mouse	ThermoFisher Scientific	A-21200	1/200
DyLight 594	Goat anti-hamster (Armenian)	BioLegend	405504	1/250
Alexa Fluor 594	Donkey anti-Rabbit	ThermoFisher Scientific	R37119	2 drops/ mL
Alexa Fluor 555	Goat anti-Rabbit	ThermoFisher Scientific	A-21428	1/200
Alexa Fluor 546	Donkey anti-Goat	ThermoFisher Scientific	A-11056	1/200

Supplementary results

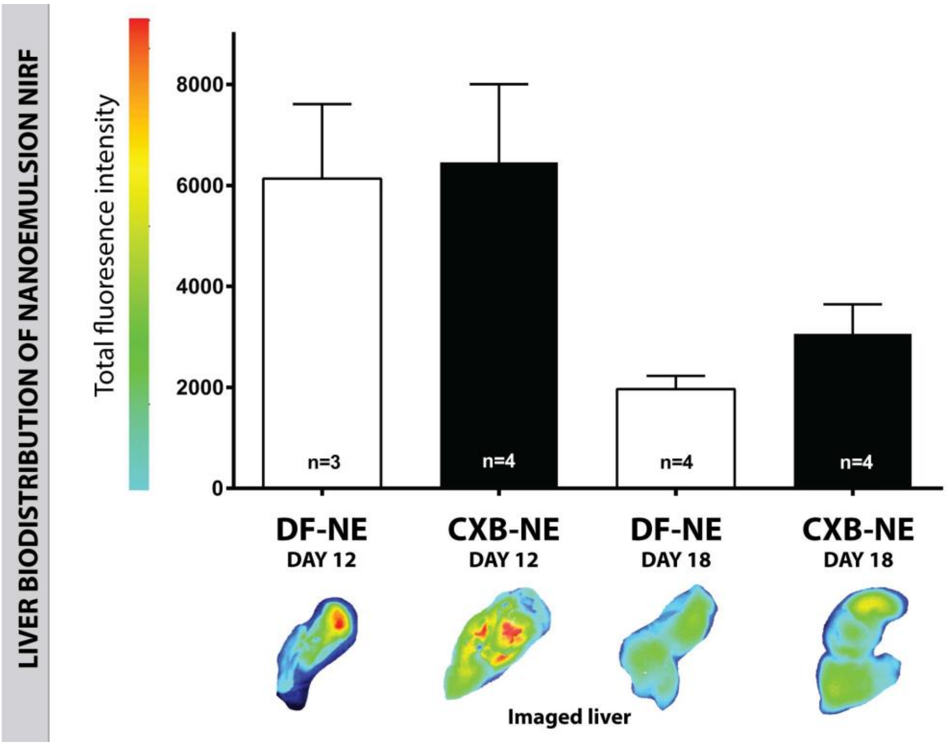

Supplementary Figure S1. Clearance of NIRF signal from the liver.

The relative NIRF signal was measured in a preclinical fluorescence imager to evaluate the biodistribution of nanomedicine in the liver. Total fluorescence intensity was measured and analyzed by one-way ANOVA, with a Tukey's post hoc test, however there was no statistical significance between testing groups. There does however appear to be a trend in both the DF-NE and CX-NE groups showing an approximately 2-fold decrease in NIRF signal, suggesting that NIRF signal has been cleared from the liver over time. A color representation of total fluorescence intensity is presented: the color scale ranges from cyan (low) to red (high).

References

1. Vasudeva K, Andersen K, Zeyzus-Johns B, Hitchens TK, Patel SK, Balducci A, et al. Imaging neuroinflammation in vivo in a neuropathic pain rat model with near-infrared fluorescence and (1)(9)F magnetic resonance. PLoS One [Internet]. 2014/03/04. 2014;9:e90589. Available from: https://www.ncbi.nlm.nih.gov/pubmed/24587398

2. Janjic JM, Vasudeva K, Saleem M, Stevens A, Liu L, Patel S, et al. Low-dose NSAIDs reduce pain via macrophage-targeted nanoemulsion delivery to neuroinflammation of the sciatic nerve in rat. J Neuroimmunol [Internet]. 2018;318:72–9. Available from: https://doi.org/10.1016/j.jneuroim.2018.02.010
